# Macrophages and Dendritic Cells as Actors in the Immune Reaction of Classical Hodgkin Lymphoma

**DOI:** 10.1371/journal.pone.0114345

**Published:** 2014-12-03

**Authors:** Christiane Silke Tudor, Heiko Bruns, Christoph Daniel, Luitpold Valentin Distel, Arndt Hartmann, Armin Gerbitz, Maike Julia Buettner

**Affiliations:** 1 Department of Nephropathology, Institute of Pathology, University Hospital Erlangen, Friedrich-Alexander-University Erlangen-Nürnberg, 91054 Erlangen, Germany; 2 Department of Hematology and Oncology, University Hospital Erlangen, Friedrich-Alexander-University of Erlangen-Nürnberg, University Hospital, 91054 Erlangen, Germany; 3 Department of Radiation Oncology, University Hospital Erlangen, Friedrich-Alexander-University of Erlangen-Nürnberg, University Hospital, 91054 Erlangen, Germany; 4 Institute of Pathology, University Hospital Erlangen, Friedrich-Alexander-University Erlangen-Nürnberg, 91054 Erlangen, Germany; IRCCS National Cancer Institute, Italy

## Abstract

**Background:**

The inflammatory infiltrate plays a pivotal role in classical Hodgkin lymphoma (cHL). Here, we focussed on the role of macrophages (MΦ) and dendritic cells (DC).

**Methods:**

MΦ and DC infiltration was investigated in 106 cHL specimens using immunohistochemistry and cytokine expression was analyzed in a subset by real-time PCR. Human peripheral blood-derived monocytes, DC, MΦ stimulated with GM-CSF (MΦ^GM-CSF^, pro-inflammatory MΦ-1-model) or M-CSF (MΦ^M-CSF^, immunomodulatory MΦ-2-model) were incubated with cHL cell line (L1236, HDLM2) supernatants (SN). DC maturation or MΦ polarization were investigated by flow cytometry. Furthermore, the impact of DC or MΦ on cHL cell proliferation was analyzed by BrdU/CFSE assay.

**Results:**

In cHL tissues mature myeloid (m)DC and MΦ predominated. High numbers of CD83+ mDC and low numbers of CD163+ MΦ were associated with improved disease specific survival. In numerous cHL specimens increased levels of both pro- and anti-inflammatory cytokines and of IL13 and GM-CSF were observed compared to reactive lymphadenopathies. Maturation of DC and induction and maintenance of an immunomodulatory MΦ phenotype were promoted by SN derived from cHL cell lines. TNFα neutralization in SN resulted in a significant inhibition of mDC maturation. DC and pro-inflammatory MΦ inhibited the proliferation of cHL cells.

**Conclusion:**

Adopting an immunomodulatory phenotype is a potential mechanism for how MΦ promote immune evasion in cHL. Mature DC, in contrast, might participate in antitumoral immunity.

## Introduction

Intense attention has been paid to antigen-presenting cells (APC) as part of the tumor stroma. Myeloid dendritic cells (mDC) are found in different malignancies [1] and evidence exists that plasmacytoid DC (pDC) may have a tolerogenic intratumoral function [2], [3]. Among tumor-infiltrating APC tumor-associated macrophages (TAM) predominate [4], [5].

In classical Hodgkin lymphoma (cHL) few malignant Hodgkin-Reed-Sternberg (HRS) cells are embedded in a prominent inflammatory infiltrate [6], [7]. The occurrence of DC in cHL has been reported and data on a potential influence on the prognosis of cHL patients were ambiguous [8]–[10]. Studies in breast cancer implicated that the tumor modulates DC maturation [11]. An inhibitory effect on DC maturation by tumoral production of mediators like IL10, TGFβ and M-CSF has been proposed [1]. Moreover, in diverse tumors a beneficial effect of DC on clinical outcome was demonstrated [12]–[14].

Immune evasion might also be achieved by modulating the polarization of intratumoral macrophages (MΦ), being numerous in HL [15]. The tumor might promote their polarization towards an anti-inflammatory phenotype characterized by low cytotoxicity and low expression of inflammatory cytokines [16]–[19]. TAM and neoplastic cells produce diverse factors including IL10, TGFβ, TNFα, PGE2 and IL1 known to promote tumor progression, induce regulatory T cells, suppress cytotoxic T and NK cells and influence DC maturation and MΦ polarization [18], [20]–[24]. In most studied malignancies the excessive presence of TAM was a poor prognosticator [4], [20]. A tumor-promoting role of TAM in cHL first suggested decades ago was confirmed by gene expression profiling and immunohistochemical studies recently [15], [25]–[30]. TAM have been reported to show an intermediate phenotype that can exert pro-inflammatory/antitumoral functions under normoxic conditions [16], [31] besides anti-inflammatory/protumoral functions, which might explain that other groups found no association of MΦ numbers and survival in cHL [32]–[34].

In the present study we aimed to investigate the role of mDC and MΦ in cHL cases by analyzing their distribution and influence on outcome as well as the intratumoral cytokine profile. Furthermore, in a cell culture system the mutual effect of cHL cell lines on monocytes, monocyte-derived (mo)DC and MΦ and vice versa was investigated.

## Methods

### cHL tissue specimens

Formalin-fixed paraffin-embedded (FFPE) specimens of 106 cHL cases and of 10 reactive lymphadenopathies were retrieved from the Archives of the Institute of Pathology, University Hospital, Erlangen, Germany. Most cases have been included in a previous study [35]. An overview of the clinical data is provided in Table 1.

**Table 1:** Overview of the clinical data.

Characteristic	n(%)
**cHL cases**	106 (100)
nsHL	65 (61.32)
mcHL	38 (35.85)
Others	3 (2.83)
**Gender**	
Male	66 (62.26)
Female	40 (37.74)
**EBV status**	
EBV negative	68 (64.15)
EBV positive	36 (33.96)
Not known	2 (1.89)
**Ann-Arbor**	
Stage I	16 (15.09)
Stage II	43 (40.57)
Stage III	25 (23.59)
Stage IV	13 (12.26)
Stage not known	9 (8.49)
**Age at diagnosis (years)**	38.92±20.02
**Follow up (years)**	7.78±4.91
**Tumor death**	11 (10.38)

The use of FFPE material from the Archive of the Institute of Pathology was approved by the Ethics Committee of the Friedrich-Alexander-University of Erlangen-Nuremberg (ethic@zuv.uni-erlangen.de) on 24^th^ January 2005, waiving the need for retrospective consent for the use of archived rest material.

### Immunohistochemistry and analysis of EBV association

2 µm tissue sections were de-paraffinised in xylene and rehydrated with graded ethanol. For manual stains (CD1a, CD68, CD123) antigen-retrieval was performed in a steam cooker (Biocarta Europe, Hamburg, Germany) for up to 5 min at 120°C using a target retrieval solution pH 6 (TRS6, Dako Cytomation, Hamburg, Germany) or 0.01 M Na-Citrate buffer pH 6. CD163 and CD83 stains were performed on a Ventana BenchMark Ultra stainer (Roche, Grenzach-Wyhlen, Germany) using CC1 buffer (Benchmark ULTRA CC1, Roche) for antigen retrieval. Primary antibodies specific for CD1a (1∶2, mouse, monoclonal, Clone O10, Cat PNIM1590, Immunotech, Beckman Coulter, Marseille, France), CD83 (1∶25, mouse, monoclonal, Clone 1H4b, Cat ab49324, abcam, Cambridge, UK), CD123 (1∶100, mouse, monoclonal, Clone 7G3, Cat 554527, BD Pharmingen, Heidelberg, Germany), CD68 (1∶200, mouse, monoclonal, Clone PG-M1, Cat M0876, Dako Cytomation) and CD163 (1∶500, mouse, monoclonal, Clone 10D6, Cat NCL-CD163, Novocastra, Newcastle, UK) were applied. In manual stains an alkaline phosphatase-labeled polymer kit was used (Zytochem-Plus AP-PolymerKit, Zytomed Systems, Berlin, Germany) or the biotin-streptavidin method. Fast Red (Sigma-Aldrich, Deisenhofen, Germany) or aminoethyl carbazole (AEC, Zymed Laboratories) served as chromogens.

To assess the EBV state of CHL cases EBER-*in situ* hybridization (ISH) or latent membrane protein 1 (LMP1) specific immunohistochemistry was performed as described before [35].

### Quantitative evaluation of histologic sections

A computer assisted hot spot analysis was performed on whole block sections. Images were acquired with a standard light microscope and a CCD camera or a Mirax Midi system (Zeiss, Jena, Germany) for digital slide scanning and Mirax viewer software (Zeiss). Positive cells were manually selected by a pathologist. Numbers of positive cells per mm^2^ in the area with the densest infiltration were evaluated using the image analysis software “Count” (Biomas, Erlangen, Germany). In CD83 stains positive cells showing the morphology of neoplastic HRS cells were excluded from the analysis. For evaluation of CD123 only cells with plasmacytoid morphology were counted.

### FFPE real-time PCR of cHL biopsies

From 10 µm FFPE sections total RNA isolation and DNAse digestion were performed, using a standardized fully automated isolation method based on germanium-coated magnetic beads (XTRAKT RNA kits, STRATIFYER Molecular Pathology GmbH, Cologne, Germany) in combination with the liquid handling robot XTRAKT SL (STRATIFYER) as previously described [36] for the analysis of *TNF*, *IL10*, *TGFβ1 and CALM2* (reference gene). For the analysis of *IL4, IL13, GM-CSF, M-CSF* and *CALM2* (reference gene) RNA was isolated using Maxwell 16 LEV RNA FFPE Kit, according to the manufacturer's instructions. A probe of L1236-RNA was used as positive control and for comparison of the PCR-runs. Standard and no-template controls were included. ΔΔC_T_ qRT-PCR with probes for *CALM2* (reference gene), *TNF*, *IL10* and *TGFβ1* as well as for *IL4, IL13, GM-CSF and M-CSF* was performed with 40 cycles of nucleic acid amplification using QuantiFast Probe Assays for one-step qRT-PCR (Qiagen, Hilden, Germany). Assays were analyzed by the Applied Biosystems 7500 Fast Real-Time PCR System and the 7500 Software Version 2.0.6 (Applied Biosystems, Darmstadt, Germany).

### Cell lines and isolation of moDC and MΦ

The cHL cell lines L1236 and HDLM2 were obtained in 2009 and 2002 from “Deutsche Sammlung von Mikroorganismen und Zellkulturen”, Braunschweig, Germany. The cells showed heterogeneity in cell size and typical morphology including mono-, bi-, and also multi-nucleated cells. HDLM2 were suspension cells and L1236 semi-adherent as stated by the “Deutsche Sammlung von Mikroorganismen und Zellkulturen” and showed typical growth characteristics. Cells were negative for mycoplasma at the onset of the study and were re-tested negative at the end of the study. L1236 and HDLM2 and blood cells were maintained at 37°C and 5% CO_2_ in RPMI-1640 (Sigma-Aldrich) supplemented with 10% fetal bovine serum, 50 U/ml penicillin and 50 µg/ml streptomycin (Invitrogen, Darmstadt, Germany). PBMCs were isolated by Ficoll-Paque (GE Healthcare, Uppsala, Sweden) gradient centrifugation of peripheral blood from 40 healthy volunteers (mean age 35 years).

Monocytes were isolated by plastic adherence. For generation of immature moDC, adherent cells after 2 h at 37°C were incubated with 10 ng/ml rhIL4 and 25 ng/ml rhGM-CSF (CellGenix, Freiburg, Germany) for 6 days with cytokine renewal at day 2 and 5. To generate MΦ, adherent cells were incubated for 5 days with either 50 ng/ml rhGM-CSF (MΦ^GM-CSF^, a model of MΦ-1) or 50 ng/ml rhM-CSF (MΦ^M-CSF^, a model of MΦ-2). All other cytokines were also obtained from R&D Systems, Minneapolis, USA. moDC and MΦ purity and differentiation was confirmed by FACS analysis (see Figure S1). Of all volunteers written informed consent was obtained and the procedure was approved by the Ethics Committee of the Friedrich-Alexander-University of Erlangen-Nuremberg (Re.-No. 3832).

### Flow Cytometry

For surface staining moDC were incubated with FITC-conjugated mouse anti-CD40 mAB (10 µl/stain, Clone B-B20, Cat ab27281, abcam), APC-conjuagted mouse anti-CD83 mAB (10 µl/stain, Clone HB15e, Cat 551073, BD Pharmingen) and PE-conjugated mouse anti-CD14 mAB (10 µl/stain, Clone MΦP9, Cat 345785, BD Pharmingen) or isotype controls (5 µl/stain; mouse; monoclonal; FITC-IgG1, Cat ABIN118618, Antibodies-Online, Aachen, Germany; APC-IgG1, Cat 555751, BD; PE-IgG2b, Cat 555743, BD) for 15 min at 4°C and fixed in 1% PFA. MΦ were fixed and permeabilized using the Cytofix/Cytosperm kit (BD Pharmingen) and were then incubated with FITC-conjugated mouse anit-CD68 mAB (5 µl/stain, Clone Y1/82A, Cat 562117, BD Pharmingen), PE-conjugated mouse anit-CD163 mAB (10 µl/stain, Clone GHI/61, Cat 556018, BD Pharmingen), PerCP-conjugated mouse anti-HLA-DR mAB (10 µl/stain, Clone L243, Cat 347402, BD), and APC-conjugated mouse anti-CD206 mAB (5 µl/stain, Clone 19.2, Cat 550889, BD Pharmingen) or isotype controls (5 µl/stain; mouse; monoclonal; FITC-IgG2b, Cat 555057, BD; PE-IgG1, Cat 555749, BD; PerCP-IgG2a, Cat 349054, BD; APC-IgG1, Cat 555751, BD) for 30 min at 4°C. Flow cytometric analysis was performed on a FACSCanto II flow cytometer using the FACSDiva software v6.1.3 (BD Pharmingen). Data were analyzed using FlowJo software (Tree Star, Ashland, OR, USA).

### TNFα neutralization

moDC were incubated with L1236-SN, generated after 48 h incubation of 10^6^ cells/ml and with either 10 µg/ml neutralizing anti-TNFα mAB (mouse, Clone 28401, Cat MAB610, R&D Systems) or Infliximab (mouse mAB, Cat 00688858, Remicade, MSD Sharp & Dohme Gmbh, Haar, Germany) for 24 h.

### CFSE proliferation assay

4–8×10^6^ L1236 cells were stained with 3.5 µM CFSE (Sigma-Aldrich) for 4 min at RT. 1.5×10^5^ CFSE-labeled L1236 cells/ml were incubated for 96 h with MΦ^GM-CSF^-SN, MΦ^M-CSF^-SN or moDC-SN generated from 5×10^5^ cells/ml after 48 h incubation. FACS analysis was performed to measure the CFSE signal/cell with a reduction of intensity as a marker of proliferation.

### BrdU proliferation assay

MΦ^GM-CSF^ and MΦ^M-CSF^ were co-cultured with L1236 cells in 96-well plates at a ratio of 6∶1 for 48 h with 50% of MΦ^GM-CSF^ or MΦ^M-CSF^ conditioned medium (medium generated after 5 days of monocyte incubation with either rhGM-CSF or rhM-CSF) and 100 µM BrdU (Sigma-Aldrich). Alternatively 3×10^5^ L1236 cells/ml were treated with 90% of MΦ^GM-CSF^-SN or MΦ^M-CSF^-SN alone. After centrifugation cells were washed with PBS, air dried, fixed in ice cold methanol for 20 min and then a blocking solution (1%BSA, 10%FBS, 0.02%NaAz, PBS) was added for 1 h. The mouse anti-BrdU mAB (1∶12, Clone BU-1, Cat 05-633, Millipore, Billerica, MA, USA) was added over night, followed by an Alexa Fluor 488 coupled secondary antibody (1∶100, Invitrogen) for 1 h. Fluorescence intensity was measured at an excitation of 485/20 and an emission of 528/20 using Synergy 2 (BioTek).

### Statistical analyses

Continuous variables with normal distribution and homogenous variances were analyzed by unpaired or paired t-test. Mann-Whitney U-tests or Wilcoxon signed rank test were used for data with skewed distributions. Disease-specific survival (DSS) of cHL was defined from the receipt of tissue until the last follow-up or death caused by cHL. DSS rates of cHL patients were calculated according to Kaplan-Meier. DSS curves were compared with log-rank test. In all tests p<0.05 was accepted as statistically significant. Statistical analyses were performed using SPSS for Windows software (version 19.0 SPSS, IBM, Munich, Germany) or GraphPad Prism 5 for Windows software (version 5.02, GraphPad software Inc., San Diego, CA, USA).

## Results

### Distribution and prognostic impact of MΦ and DC in cHL

**Figure 1:**
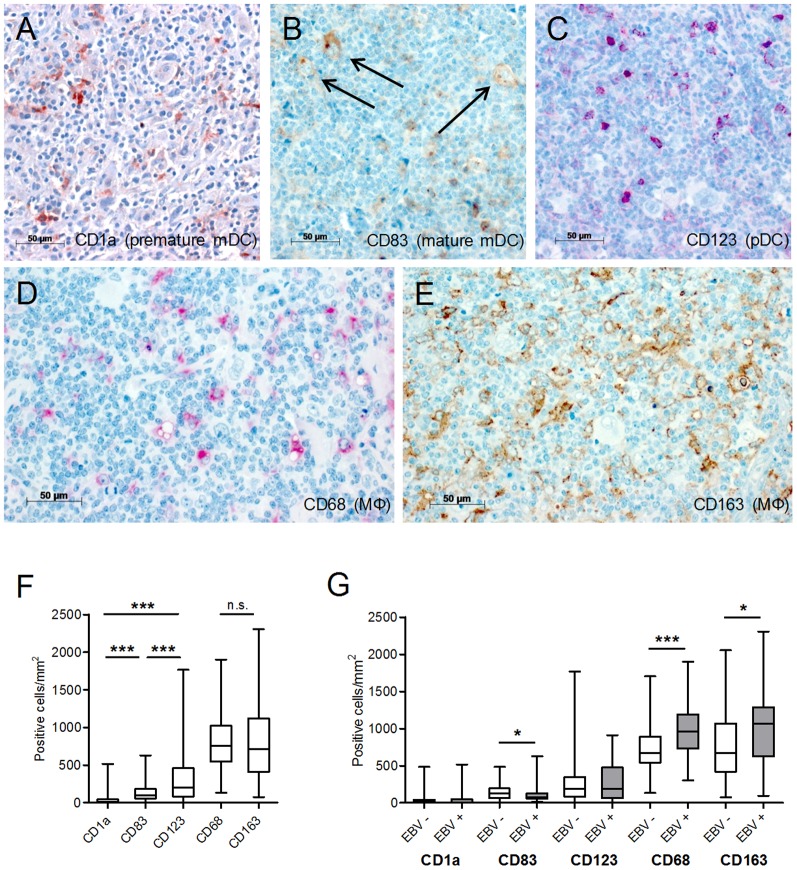
Distribution of immature and mature mDC, pDC, MΦ and CD163+ MΦ in cHL. (A) CD1a staining (n = 101) with numerous small reactive tumor infiltrating DC besides few CD1a-negative HRS cells. (B) CD83 staining (n = 97) with small infiltrating DC besides CD83-positive HRS cells (arrow). (C) CD123 staining (n = 97) showing pDC with a plasmacytoid morphology besides CD123 negative HRS cells. (D) CD68 staining (n = 96) with a cytoplasmatic positivity of MΦ and (E) CD163 staining (n = 94) with a membranous and cytoplasmic positivity of TAM. Examples of cHL specimens are depicted all acquired with a Zeiss Imager.A1 (400x magnification, 40x/0.75 objective) using AxioVision SE64 Rel.4.8 software. (F) Box-Plots show the distribution of the different DC subtypes and MΦ in the investigated cHL cases. (G) Comparison of the distribution of DC and MΦ in EBV- and EBV+ cHL. Unpaired student's t-tests or Mann-Whitney-U-tests were conducted with *P<0.05, **P<0.01 and ***P<0.001. Data in (F) are indicated as Box-and-Whisker Plots with median. Data in (G) are indicated as mean with SEM.

CD1a and CD83 were used as markers of immature and mature mDC as described elsewhere [1] (Figure 1 A and B). In cHL the majority of mDC could be identified as mature CD83+ mDC and a significantly minor portion as CD1a+ immature mDCs (Figure 1 F). CD123+ pDC were the most numerous DC type in cHL (Figure 1 C and F). For the quantification of MΦ, the lysosomal marker CD68 as a pan- MΦ marker and CD163, a marker strongly expressed on immunomodulatory MΦ, were applied (Figure 1 D and E). MΦ were by far the most prominent cell type. Detection of nearly similar amounts of CD68+ MΦ and CD163+ MΦ implicated that the majority of MΦ show some characteristics of immunomodulatory MΦ in cHL (Figure 1 F). EBV+ cases showed more MΦ (CD68 and CD163) and fewer mature mDC than EBV- cases (Figure 1 G).

**Figure 2:**
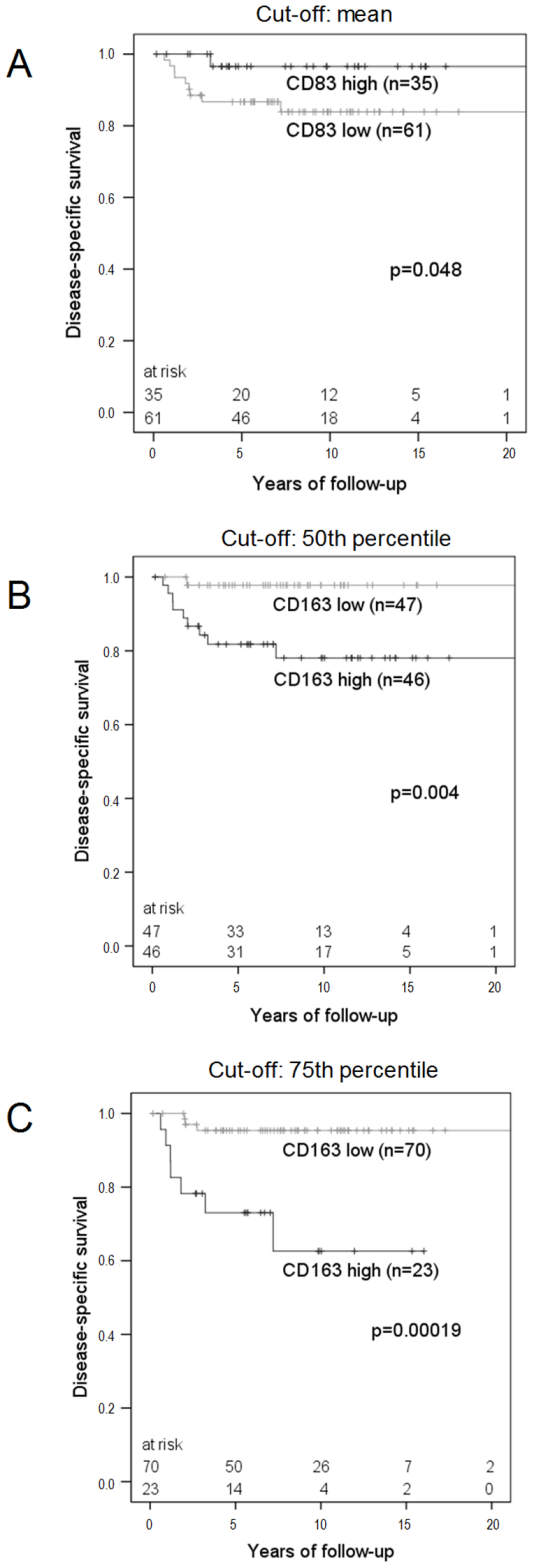
Influence of mature mDC and CD163+ MΦ on the clinical outcome of cHL patients. Kaplan-Meier-Analysis of disease-specific survival (DSS) of cHL patients with regard to the numbers of infiltrating CD83+ mature mDC (A) and CD163+ MΦ (B, C). (A) The mean and (B) the 50^th^ percentile and (C) the 75^th^ percentile were used as cut-off, to define the groups with high and low numbers of CD83+ cell/mm^2^ and CD163+ cell/mm^2^.

In Kaplan-Meier analysis the numbers of CD1a+, CD68+ and CD123+ cells did not influence DSS of cHL (cut-off median: p = 0.39, p = 0.10 and p = 0.25). DSS of patients with high numbers of CD83+ mature mDC was significantly improved (Figure 2 A). Conversely high numbers of CD163+ cells were strongly associated with reduced DSS (Figure 2 B and C).

### Cytokine profile in cHL specimens

**Figure 3:**
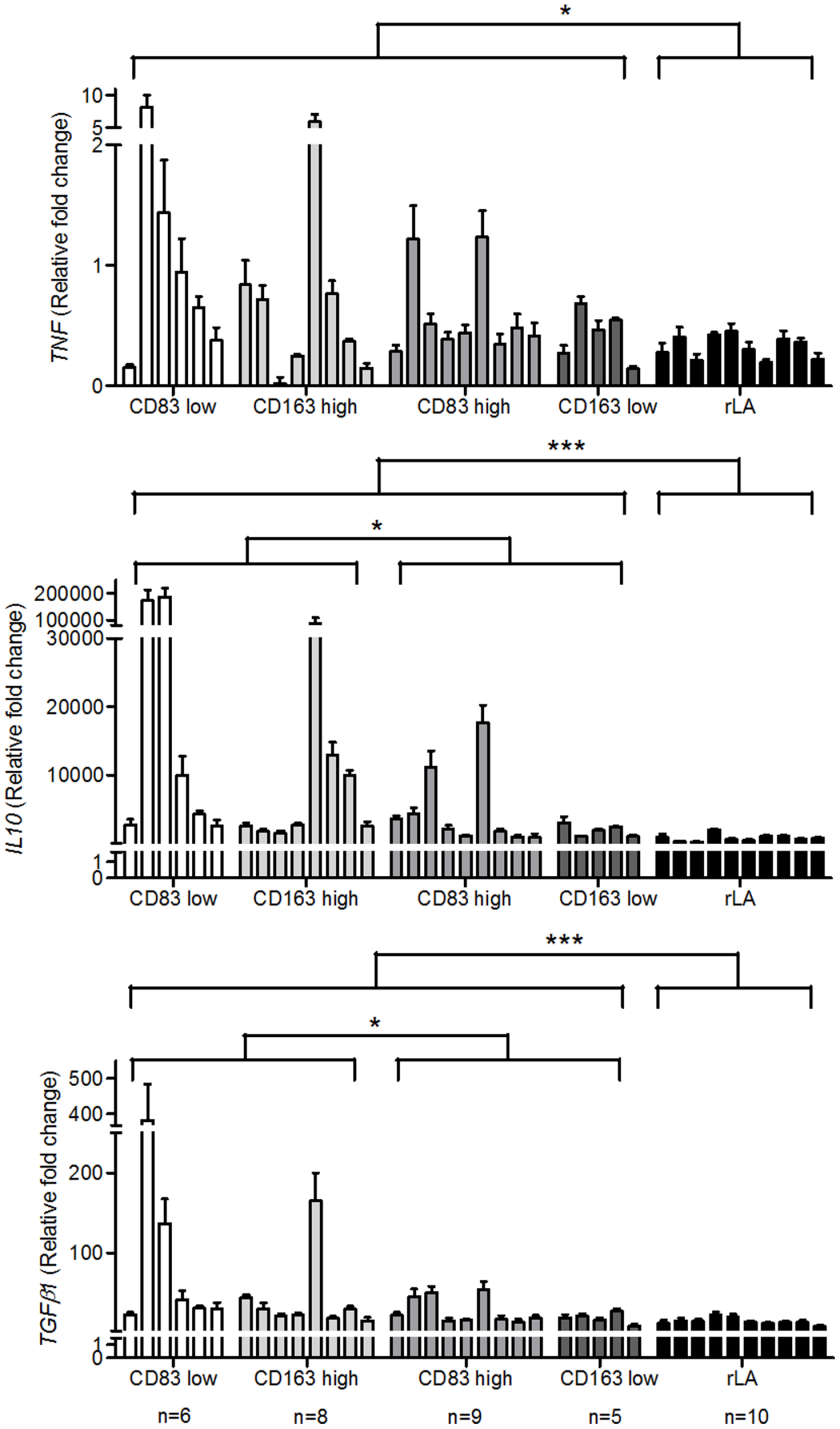
Expression of *TNF*, *IL10* and *TGFβ1* in cHL cases with high or low numbers of mature mDC and CD163+ MΦ. RNA was isolated from FFPE cHL sections and subjected to qRT-PCR. The expression of *TNF, IL10* and *TGFβ1* was determined. cHL cases were grouped into subgroups with low or high numbers of CD83+ or CD163+ infiltrating cells using the 25. or 75. percentile as cut-off, respectively and compared to a control group of benign reactive lymphadenopathy (rLA). All values are shown as fold change compared to the measured cytokine expression in the L1236 cell line. Mann-Whitney-U-tests were conducted with *P<0.05, **P<0.01 and ***P<0.001 comparing either all cHL cases with rLA or cHL cases with presumably adverse prognosis (CD83 low and CD163 high) with those with an expected better prognosis (CD83 high and CD163 low). Single bars represent the mean + SD of triplicates of single analyzed cases, each.

**Figure 4:**
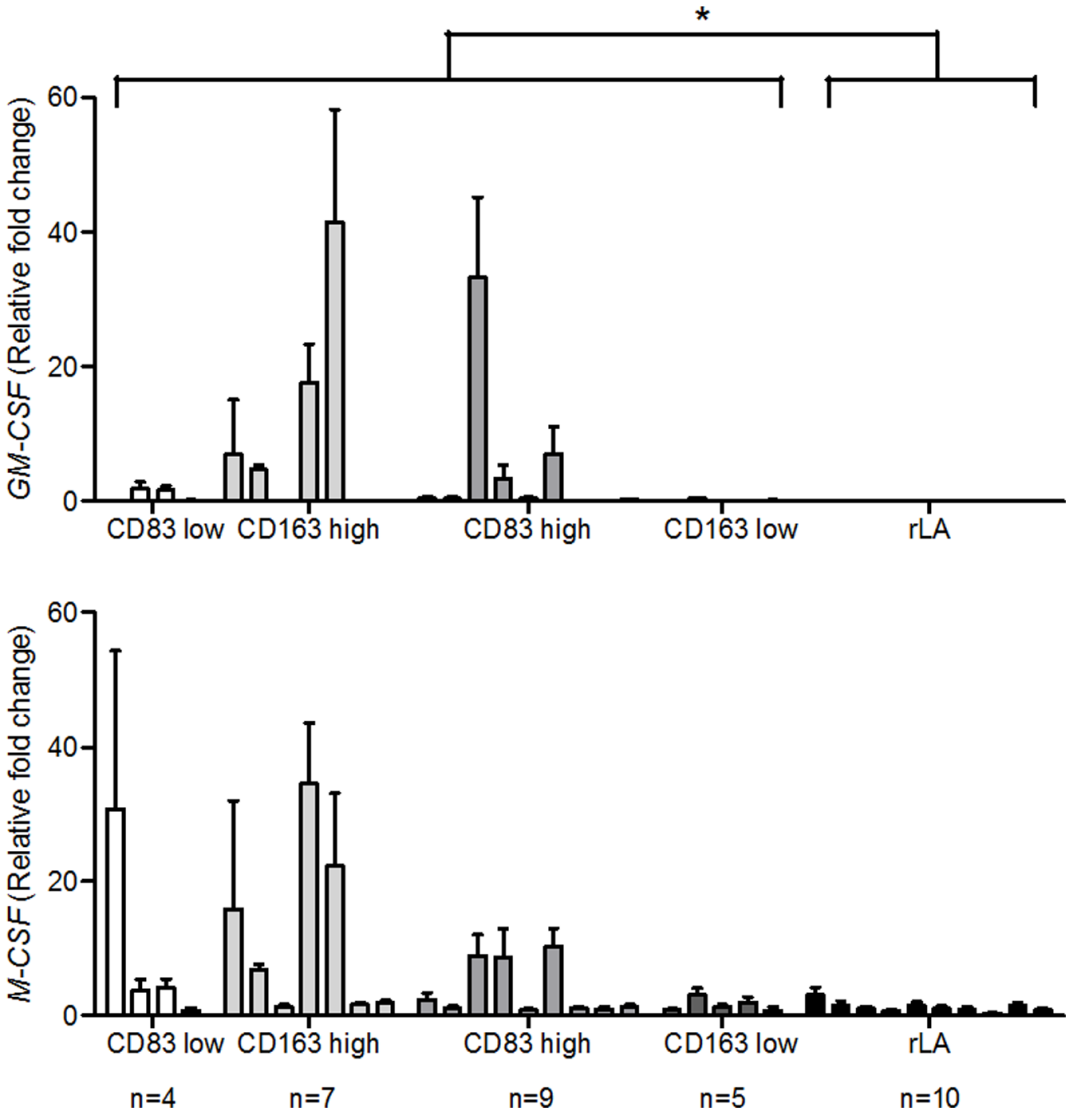
Expression of *GM-CSF* and *M-CSF* in cHL cases with high or low numbers of mature mDC and CD163+ MΦ. RNA was isolated from FFPE cHL sections and subjected to qRT-PCR. The expression of *GM-CSF* and *M-CSF* was determined. cHL cases were grouped into subgroups with low or high numbers of CD83+ or CD163+ infiltrating cells using the 25. or 75. percentile as cut-off, respectively and compared to a control group of benign reactive lymphadenopathy (rLA). All values are shown as fold change compared to the measured cytokine expression in the L1236 cell line. Mann-Whitney-U-tests were conducted with *P<0.05, **P<0.01 and ***P<0.001 comparing either all cHL cases with rLA or cHL cases with presumably adverse prognosis (CD83 low and CD163 high) with those with an expected better prognosis (CD83 high and CD163 low). Single bars represent the mean + SD of triplicates of single analyzed cases, each.

**Figure 5:**
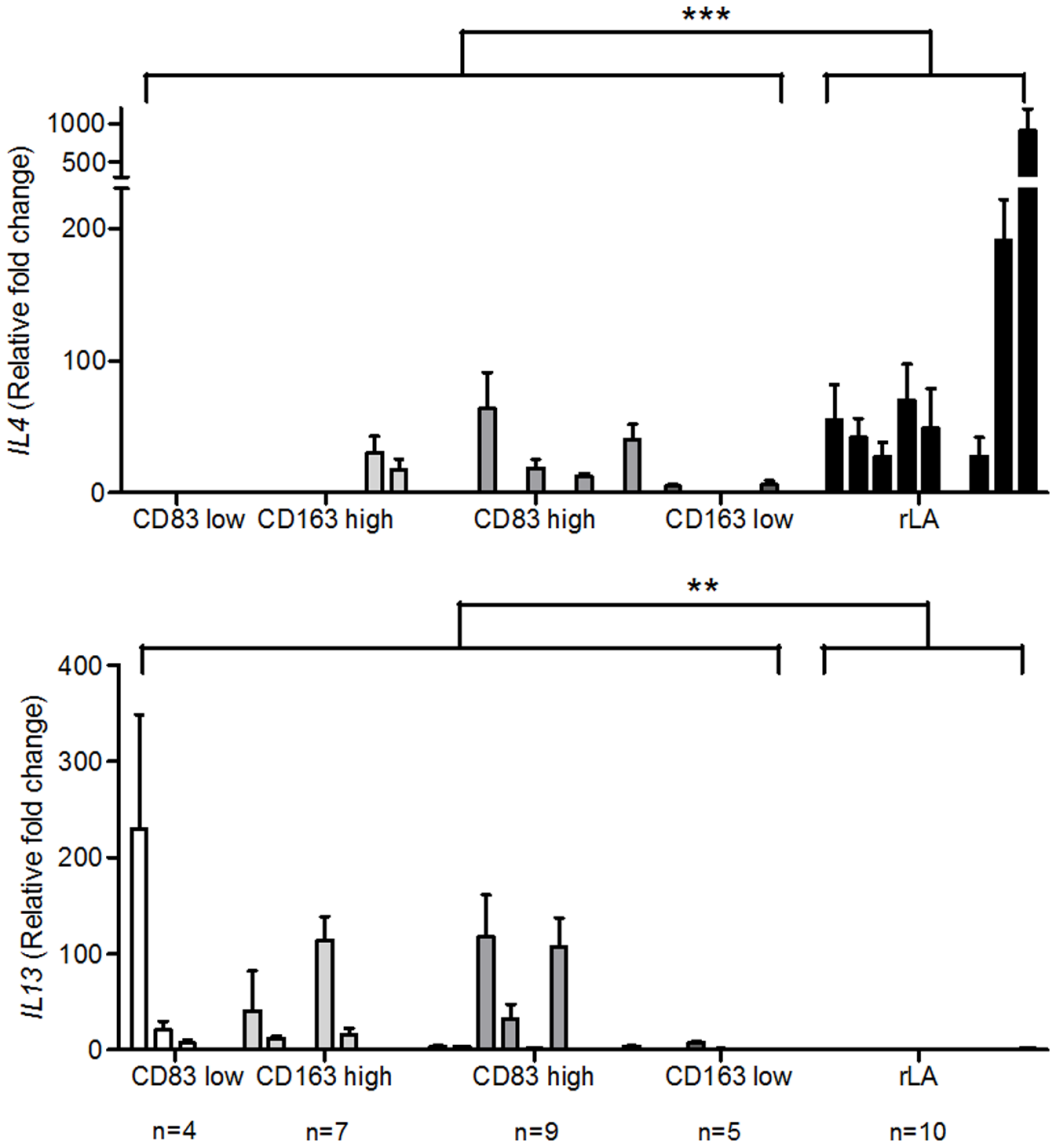
Expression of *IL4* and *IL13* in cHL cases with high or low numbers of mature mDC and CD163+ MΦ. RNA was isolated from FFPE cHL sections and subjected to qRT-PCR. The expression of *IL4* and *IL13* was determined. cHL cases were grouped into subgroups with low or high numbers of CD83+ or CD163+ infiltrating cells using the 25. or 75. percentile as cut-off, respectively and compared to a control group of benign reactive lymphadenopathy (rLA). All values are shown as fold change compared to the measured cytokine expression in the L1236 cell line. Mann-Whitney-U-tests were conducted with *P<0.05, **P<0.01 and ***P<0.001 comparing either all cHL cases with rLA or cHL cases with presumably adverse prognosis (CD83 low and CD163 high) with those with an expected better prognosis (CD83 high and CD163 low). Single bars represent the mean + SD of triplicates of single analyzed cases, each.

For the analyses of RNA isolated from cHL specimens, cases were selected with numbers per mm^2^ of CD83+ or CD163+ cells below (low) or above (high) the 25^th^ or 75^th^ percentile, respectively. *TNF, IL10*, *TGFβ1* were investigated, which were reported in cHL tissue and can influence DC maturation/development and MΦ polarization [1], [7], [23], [24]. Additionally, *GM-CSF, M-CSF, IL4* and *IL13* were analyzed, which might also partake in the polarization of MΦ [17], [37]. Because of the degradation of RNA in FFPE material specifically designed taqman probes for sequences <100 bp were applied. Results were normalized to RNA isolated from L1236 cells and were compared to reactive lymphadenopathies. Cases expected to have an inferior outcome, namely CD83 low and CD163 high, showed a significantly higher expression of *IL10* and *TGFβ1* compared to the other cHL cases (Figure 3). A significantly induced expression of *TNF, IL10, TGFβ1, GM-CSF and IL13* was detected in cHL compared to rLA (Figures 3 to 5). The only cytokine which was found predominantly in rLA compared to cHL was *IL4* (Figure 5).

### Influence of cytokines and cHL cell line SN on the maturation of moDC and polarization of peripheral blood monocytes and MΦ

**Figure 6:**
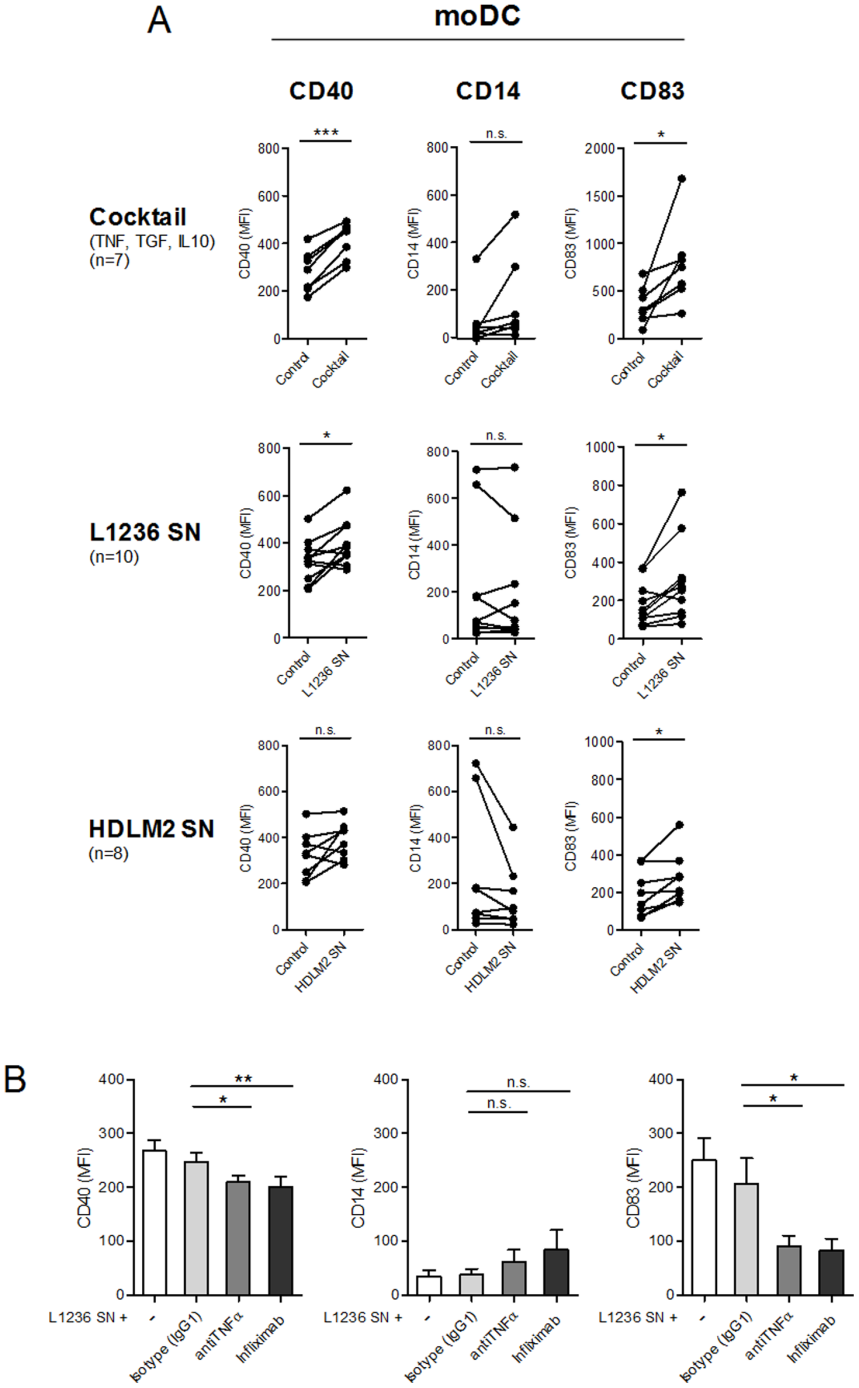
Characterization of the maturation and activation of moDC. (A) moDC were treated for 24 h with TNFα, IL10 and TGFβ (each 10 ng/ml), 50% L1236 or HDLM2 supernatants (SN) or with (B) 50% L1236 supernatants and either an IgG1 isotype control, an neutralizing TNFα antibody or a therapeutically applied TNFα antibody (infliximab). Supernatants were generated at a concentration of 1×10^6^ cells/ml medium after 48 h incubation. The expression of the DC activation marker CD40, the monocyte marker CD14 and the DC maturation marker CD83 was determined by flow cytometry. Mean fluorescence intensity (MFI) is indicated on the bar charts. Paired student's t-tests or Wilcoxon signed-rank tests were conducted with *P<0.05, **P<0.01 and ***P<0.001.

Cytokines known to influence DC maturation and to be expressed in cHL [1], [23] were selected to validate the experimental system. As anticipated, TNFα induced the activation marker CD40 and the maturation marker CD83. IL10 counteracted moDC development as shown by the upregulation of the monocyte marker CD14. In addition, moDC maturation was blocked by TGFβ downregulating CD83 (Figure S2 A). To reflect at least in part the diversity of immune mediators found in cHL, moDC were treated with a cytokine cocktail (TNFα/IL10/TGFβ) or with SN of cHL cell lines L1236 and HDLM2. All treatments induced significant moDC activation and/or maturation similar to TNFα alone (Figure 6 A and Figure S2 A). This effect was more pronounced in L1236-SN with higher TNFα concentrations than HDLM2-SN (Figure S2 B). When incubating moDC with L1236-SN and neutralizing TNFα antibodies a significant reduction of CD40 and CD83 upregulation was observed (Figure 6 B). SN generated from B-cells from PBMC as a control showed no effect on moDC when compared to medium controls (Figure S2 A).

**Figure 7:**
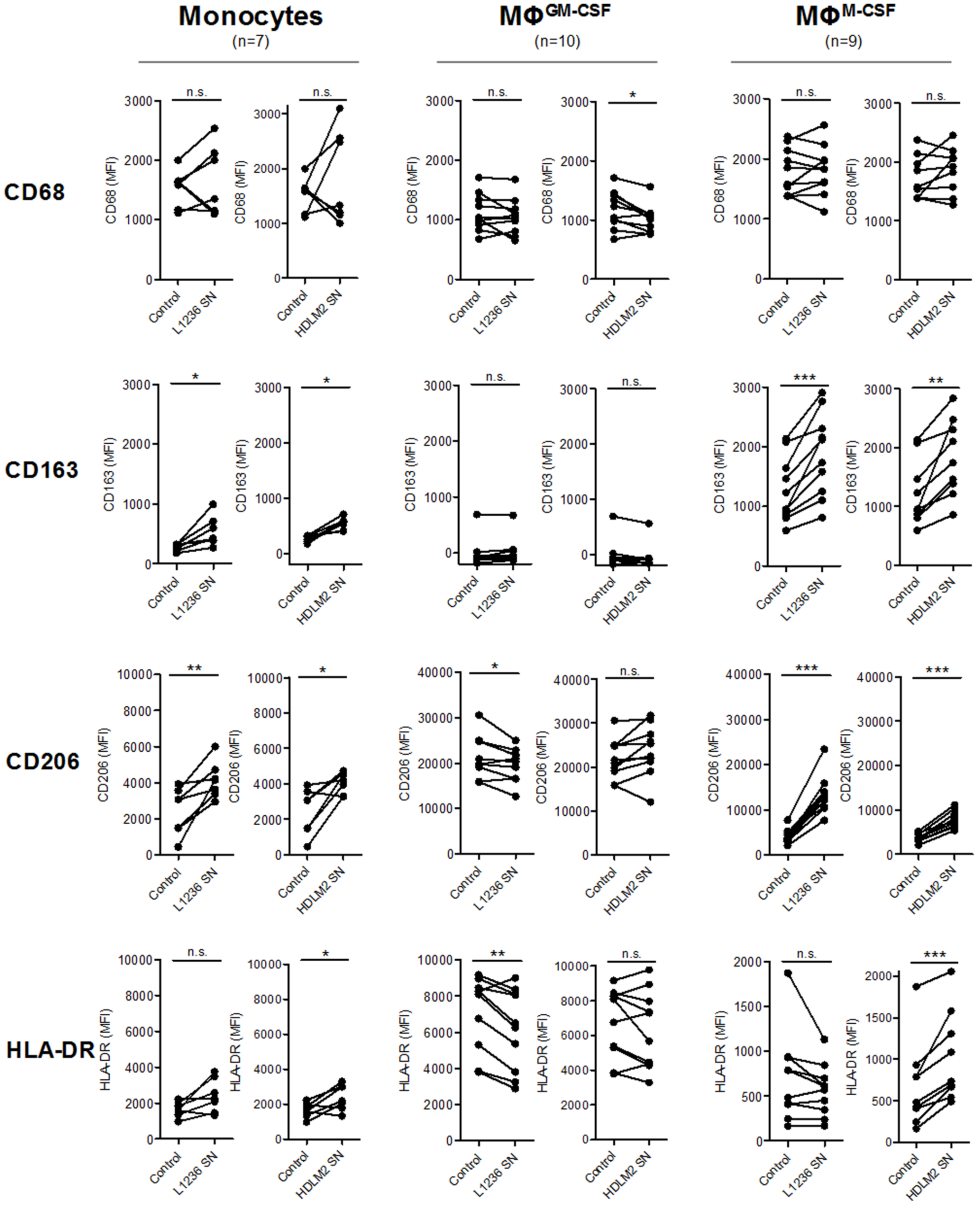
Characterization of the polarization profile of monocytes, MΦ^GM-CSF^ (MΦ-1 model) and MΦ^M-CSF^ (MΦ-2 model). Monocytes were cultured for 5 days in complete medium or 50% L1236 or HDLM2 SN. MΦ^GM-CSF^ and MΦ^M-CSF^ were treated for 24 h with medium or 50% L1236 or HDLM2 SN. The expression of different MΦ lineage markers was determined, including CD68, CD163, CD206 and HLA-DR. Mean fluorescence intensity (MFI) is indicated on the bar charts. Paired student's t-tests or Wilcoxon signed-rank tests were conducted with *P<0.05, **P<0.01 and ***P<0.001.

MΦ^GM-CSF^ (MΦ -1 model) expressed high levels of HLA-DR and lacked CD163 expression whereas MΦ^M-CSF^ (MΦ-2 model) presented with high CD163 and low HLA-DR expression, as expected (Figure 7). To address the question whether MΦ polarization could be modulated by cHL-conditioned medium, monocytes, MΦ^GM-CSF^ and MΦ^M-CSF^ were incubated with L1236-SN and HDLM2-SN. Changes in the expression of CD68, a pan MΦ marker, the markers scavenger receptor CD163 and mannose receptor CD206, which are particularly found on immunomodulatory MΦ and of HLA-DR predominantly expressed by pro-inflammatory MΦ were analyzed (Figure 7). In MΦ^M-CSF^ and monocytes a consistent induction of CD163 and CD206 upon treatment with L1236-SN and HDLM2-SN was seen, whereas the SN had no effect on CD163 expression in MΦ^GM-CSF^. CD206 was slightly down-regulated by L1236-SN in MΦ^GM-CSF^ but not by HDLM2-SN.

### Modulation of the proliferative activity of cHL cells by moDC and MΦ

**Figure 8:**
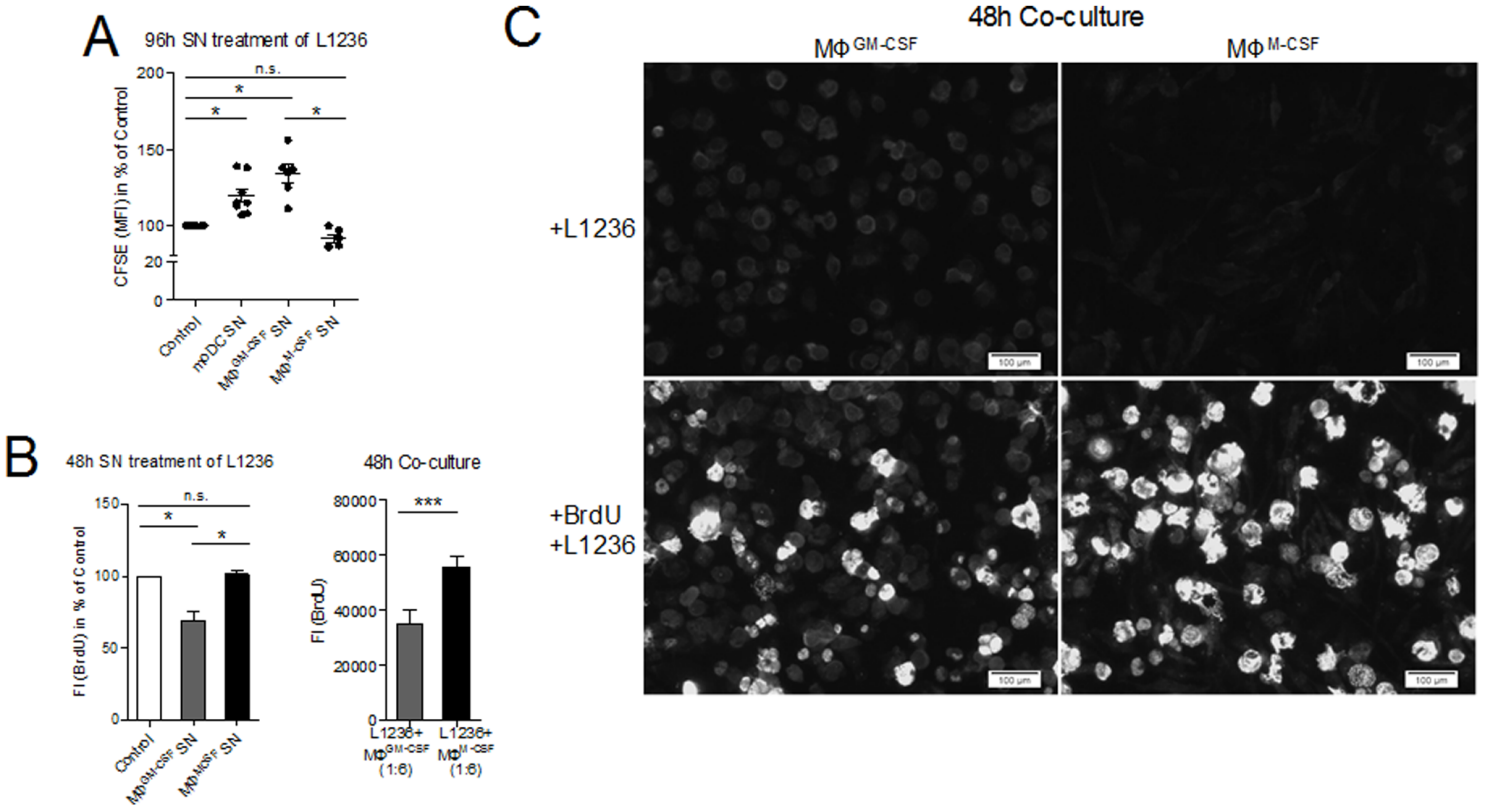
Effects of moDC, MΦ^GM-CSF^ and MΦ^M-CSF^ on the proliferation of L1236 cells. (A) L1236 cells were incubated with 90% moDC-, MΦ^GM-CSF^ - or MΦ^M-CSF^ - supernatants in the presence of CFSE for 96 h. Supernatants were generated at a concentration of 5×10^5^ cells/ml incubated for 48 h. Mean fluorescence intensity (MFI) is indicated on the bar charts as determined by flow cytometry. (B) L1236 cells were incubated with MΦ^GM-CSF^ - or MΦ^M-CSF^ -supernatants or (B and C) co-cultured at a ratio of 6∶1 in the presence of BrdU for 48 h. BrdU was stained using a BrdU-specific primary antibody and an Alexa Fluor 488-coupled secondary antibody. Fluorescence intensity (FI) was measured by a fluorescence reader. All micrographs in (C) were acquired with an Olympus IX81 (100x magnification, 10x/0.30 Ph1 objective) using cellSens dimension software. Paired student's t-tests, Wilcoxon signed-rank tests or Mann-Whitney-U-tests were conducted with *P<0.05, **P<0.01 and ***P<0.001. Data are indicated as mean with SEM of 6-9 (A) or 6-8 (B) independent experiments.

To analyze the effect of moDC and MΦ on tumor cell proliferation, CFSE stained L1236 were treated with SN of moDC, MΦ^GM-CSF^ or MΦ^M-CSF^ and CFSE signal was measured as a reciprocal indicator of cell proliferation. A significant inhibition of L1236 growth was found after incubation with moDC-SN or MΦ^GM-CSF^-SN (Figure 8 A). MΦ^M-CSF^-SN, on the contrary, did not influence L1236 growth. Similar results were obtained measuring BrdU incorporation into DNA of L1236, when treated with MΦ^GM-CSF^-SN or MΦ^M-CSF^-SN (Figure 8 B). Co-culture experiments were performed to determine by BrdU assays whether cell-cell contact enhanced the effect of MΦ on L1236 proliferation. BrdU uptake by L1236 was significantly lower when co-cultured with MΦ^GM-CSF^ compared to MΦ^M-CSF^ similar to the findings after SN treatment (Figure 8 B and C). Since co-cultures were kept in 50% MΦ-conditioned medium containing GM- or M-CSF, we excluded an effect of these cytokines on L1236 growth (Figure S3 A). Proliferation of MΦ in co-culture was assessed by Ki67-CD68 double stainings. A weak proliferative activity was observed with no significant difference between MΦ^GM-CSF^ and MΦ^M-CSF^, indicating that the difference in proliferation observed in co-culture can be assigned to L1236 (Figure S3 B).

## Discussion

In the analysis of the inflammatory infiltrate of diverse tumors close attention was paid to the role of DC and MΦ in recent years. The present study focusses on their role in cHL microenvironment as possible mediators of immune escape. To our knowledge no reports on the role of CD83+ mDC on cHL prognosis in adults exist, whereas in pediatric cHL the numbers of DC were not associated with clinical outcome [8]. The adverse prognostic impact of MΦ found in multiple malignancies, however, has been confirmed for cHL [15], [25]–[30].

In our study mature mDC outnumbered immature mDC, which indicates that HRS cells do not abolish mDC maturation as a mean of immune evasion as was suggested for breast cancer [1]. A possible explanation might be that protumoral effects of factors promoting mDC maturation like TNFα, which is also angiogenic [38], might exceed the benefits of inhibiting mDC maturation. pDC were the most frequent DC subtype in our study. MΦ were by far the most numerous population observed. The fact that the number of CD163+ cells approximated the total number of CD68+ MΦ might indicate that a very relevant subgroup of TAM has some characteristics of immunomodulatory MΦ, as CD163 in model systems is particularly strongly expressed on MΦ-2.

As reported for breast cancer [39], [40], no influence of CD1a+ immature mDC on DSS was found. Previous reports on the prognostic impact of CD1a+ cells in cHL were controversial, showing no association with outcome [9] or a poorer outcome in patients without CD1a+ cells in the tumor infiltrate [10]. In the present study, numerous CD83+ mature mDC were associated with improved outcome in cHL, as found in a variety of malignancies [1]. This could support the idea that mature DC promote antitumoral immunity by direct tumortoxicity or T cell priming. CD83 can also be expressed on pDC [41]. However, CD123+ pDC were also analyzed and had no influence on cHL outcome, as reported earlier [42]. Therefore, it appears improbable that the prognostic effect of CD83+ cells has to be attributed to an effect of pDC. As some morphological overlap with HRS cells may bias the analysis of CD83+ cells, this finding will need further confirmation in the future.

In therapy-refractory cHL a TAM-specific gene signature [15] and an association of high numbers of CD68+ MΦ with shortened progression-free survival have been reported [15]. Comparably other studies revealed that either CD163+ and/or CD68+ MΦ significantly correlated with inferior outcome [27]–[30]. Others, however, found no association of MΦ numbers and survival in cHL [32]–[34]. Here, we confirm a highly significant association of high numbers of CD163+ MΦ with inferior DSS, whereas CD68+ MΦ had no impact on outcome. CD163+ and CD68+ MΦ were more frequent in EBV+ compared to EBV- cHL, confirming previous findings [8], [27], [28], [30], [33]. This, however, was not associated with worse DSS of EBV+ cases (cut-off median: p = 0.06). Some explanations for the predominance of MΦ in EBV+ cHL were discussed. In pediatric cHL it was proposed that an antiviral immune reaction might result in an increased recruitment of both cytotoxic T cells and MΦ [8]. This would go in line with the report of a Th1 and antiviral gene profile in EBV+ cHL [42]. Moreover, monocytes were shown to induce proliferation and LMP1 expression in cells of EBV-associated nasal natural killer/T cell lymphoma [43] and a correlation of serum CD163 and plasma EBV-DNA in cHL was reported [44]. These findings might indicate a mutually inductive interaction of the virus and monocytes/MΦ. The exact mechanism, leading to an accumulation of MΦ in EBV+ cHL is, however, so far not understood. Next we investigated the cytokine profile of the cHL microenvironment. All investigated cytokines were found at least in a subset of cHL cases. A frequent expression of IL13 and M-CSF in cHL cases has been described, whereas IL4 was only detected in small amounts, going in line with our findings [45]. Comparing cHL cases to reactive lymphadenopathies, we found significantly higher amounts of *TNF, IL10, TGFβ1, GM-CSF* and *IL13* in cHL cases. Especially when comparing presumably adverse prognostic constellations in cHL (CD83 low or CD163 high) with cHL cases with an expected better prognosis (CD83 high or CD163 low) anti-inflammatory (*IL10, TGFβ1*) cytokines were significantly upregulated in the adverse group. The upregulation of immunoregulatory cytokines *IL-10* and *TGFβ1* in cHL and especially in the prognostically adverse group goes well in line with immune evasion. At first glance, in contrast, it appears paradoxical that *TNF* and *GM-CSF* are excessively produced in cHL. However, persistent inflammation has been connected to tumorigenesis [46], [47]. Therefore, both, pro- and anti-inflammatory cytokines, might promote cHL growth. The presence of *IL13* and *M-CSF* in cHL cases might be responsible for the induction of MΦ-2-like CD163+ tumor-associated macrophages in cHL [17], [37], whereas IL4 was detected infrequently and, if at all, seems to play a minor role.

Going in line with the dominance of mature mDC in cHL specimens, SN of cHL cell lines promoted the maturation and/or activation of moDC in our study. This effect was stronger for L1236 cells producing higher TNFα concentrations than HDLM2. When combining IL10 and TGFβ that hamper DC maturation and maturation-inducing TNFα [23], all known to be expressed in cHL [7], the effect of TNFα on activation and maturation predominated. Moreover, neutralization of TNFα in L1236 SN decreased the observed effects on the activation and maturation of moDC, underlining the importance of TNFα.

For the investigation of MΦ, we used a published model representing two extreme polarization states of pro-inflammatory MΦ-1 and immunoregulatory MΦ-2 obtained by stimulation with M-CSF and GM-CSF [37], which we called MΦ^GM-CSF^ or MΦ^M-CSF^ depending on the used stimulus. One has, however, to keep in mind that depending on the stimulus used, many different activation states can be achieved far beyond the MΦ-1 and MΦ-2 model [48]. So obviously this model cannot fully reflect the intermediate phenotype described for TAM [16], [31]. However, it can add to our understanding of possible interactions of polarized, circulating MΦ entering the tumor milieu with tumor cells. Incubating unstimulated peripheral blood derived monocytes with cHL SN alone sufficed to induce a MΦ-2-like phenotype with up-regulation of CD163 and CD206. Moreover, treatment with cHL cell SN conserved and enhanced the MΦ-2 phenotype in MΦ^M-CSF^, as also demonstrated by the upregulation of CD163 known to promote an anti-inflammatory microenvironment [49] and of CD206, which acts pro-angiogenic [50] and thus might promote tumor growth. cHL cell SN were, however, not able to convert MΦ-1 into MΦ-2 as a mean to debilitate potentially tumor-toxic effector cells. Soluble mediators which could be responsible for the promotion of an MΦ-2 phenotype in pre-differentiated MΦ and monocytes are IL4, IL13 and M-CSF [17]. Whereas IL4 was reported to be lacking or only very mildly expressed in L1236 [51] and HDLM2 [52], IL13 was shown to be expressed in both cell lines [45], [53], [54]. Therefore, IL13 is a good candidate for the MΦ-2-inducing effect of cHL cell SN. Moreover, the expression of M-CSF was described in cHL cells [55], [56] and we could confirm this finding on RNA-level in L1236 (data not shown), so that M-CSF might also participate. The *in vitro* finding, that MΦ-2 markers were enhanced by cHL cell SN, together with the predominance and prognostic role of CD163+ cell in cHL specimens, might therefore indicate that immunoregulatory CD163+ MΦ might play an important role in the tumor site and are actively promoted and maintained by the tumor cells. The excess of pro- as well as anti-inflammatory cytokines in the tumor tissue, however, indicates, that not a typical MΦ-2 milieu is found in the tumor, going in line with the idea that TAM cannot be assigned to one of the extreme polarization patterns of MΦ-1 or MΦ-2.

When investigating the influence of moDC and MΦ on the proliferation of the L1236 cHL cell line, we found no effect of MΦ^M-CSF^ on L1236 growth, whereas both moDC and MΦ^GM-CSF^ inhibited cHL cell proliferation. This effect was not depending on cell-cell contact. Hence, soluble factors appeared sufficient to mediate some inhibitory properties. Going in line with our observations, Dufresne et al reported that MΦ-1 inhibited the proliferation of human bladder carcinoma cells [57]. GM-CSF stimulated monocytes, our model of MΦ-1 [58], [59], and immature DC (here moDC) [60], [61] were both shown to exert cytotoxic effects on tumor cell lines. It was demonstrated that both cell types can act via soluble mediators, although cell membrane-bound mediators appear to be the major players [59], [60], [62], and some functional role of secreted TNF was implicated for both [59], [61], [62]. Whereas in one study, however, TNFalpha, TRAIL and FasL were not up-regulated in GM-CSF-stimulated human MΦ [63], in another the cytotoxic effect of SN of GM-CSF stimulated monocytes was attenuated by administration of TNF-neutralizing antibodies [59]. Moreover, GM-CSF was shown to augment the release of H_2_O_2_, another potentially cytotoxic agent, from mouse macrophages [64]. However, as M-CSF had the same effect [64], it appears improbable that this could explain the difference in cytotoxicity observed in the present study between MΦ-1 and MΦ-2. Possibly other so far not characterized substances might also be operational in the antitumoral effect observed. In contrast to our findings in human bladder cancer MΦ-2 not only tolerated but even induced tumor cell growth [57] going in line with the observation that TAM density in breast cancer correlates positively with proliferative tumor activity [65].

In conclusion, mature mDC, which associated with improved outcome in cHL patients, might partake in the antitumoral immunity either by antigen presentation or by direct cytotoxicity. CD163+ MΦ, in contrast, were associated with inferior survival and might actively be induced and maintained by the tumor cells. However, no typical immunosuppressive profile but rather an excess of anti- and pro-inflammatory cytokines was observed in cHL patients with adverse prognostic markers.

## Supporting Information

Figure S1 **Generation of moDC, pro-inflammatory MΦ^GM-CSF^ and anti-inflammatory MΦ^M-CSF^.** (A) Human peripheral blood monocytes were differentiated into moDC, MΦ^GM-CSF^ or MΦ^M-CSF^ in the presence of IL4 + GM-CSF (6 days), GM-CSF (5 days) or M-CSF (5 days), respectively. Depicted are monocytes one day after isolation (left), moDC with branching projections, MΦ^GM-CSF^ with a fried-egg morphology and MΦ^M-CSF^ with a spindle-shaped morphology. All images were acquired with an Olympus IX81 (100× magnification, 10x/0.30 Ph1 objective) using cellSens dimension software. (B) Purity analysis of moDC, MΦ^GM-CSF^ and MΦ^M-CSF^. moDC purity was shown by FACS analysis of CD11c, CD14, CD3 and CD20 (BD Pharmingen) expression, being 98%, 12%, 0% and <1%, respectively. MΦ purity was proven by FACS analysis of CD68, CD11b, CD3 and CD20 (BD Pharmingen). MΦ-1 were positive in 95%, 92%, <1% and <1%, MΦ-2 in 98%, 86%, <1% and <1%, respectively. Data are indicated as mean with SEM of 4 independent experiments. (C) Analysis of cytokine secretion. For the determination of IL10 and IL12 secretion DuoSet ELISAs (R&D Systems) were applied according to the manufacturer's instruction. For generation of supernatants (SN), cells were seeded at a concentration of 1×10^6^/ml+/−100 ng/ml LPS (Sigma-Aldrich) and incubated for 24 h. Absorbance was measured at 450 nm and 540 nm as reference using Synergy 2 (BioTek, Bad Friedrichshall, Germany). Data are shown as mean with SEM of 3–4 independent experiments each.
(PDF)

Figure S2 **Characterization of the maturation and activation profile of moDC and cytokine profile of cHL cell lines L1236 and HDLM2.** (A) moDC were treated for 24 h with 10 ng/ml TNFα, IL10 or TGFβ (each 10 ng/ml) or with 50% B-cell supernatants. B-cells were isolated from human blood PBMC by MACS separation (positive selection) using CD19 MicroBeads, FcR Blocking reagent and MS columns for an OctoMACS separator (MACS Miltenyi Biotec, Bergisch Gladbach, Germany). Supernatants were generated at a concentration of 1×10^6^ cells/ml medium after 48 h incubation. The expression of the DC activation marker CD40, the monocyte marker CD14 and the DC maturation marker CD83 was determined by flow cytometry. As anticipated, TNFα induced the activation marker CD40 and the maturation marker CD83, IL10 counter-acted moDC development as shown by the up-regulation of the monocyte marker CD14, and TGFβ blocked moDC maturation by down-regulation of CD83. SN generated from B-cells from PBMC showed no effect on moDC when compared to medium controls Mean fluorescence intensity (MFI) is indicated on the bar charts. Isotype controls are depicted as dotted lines. Paired student's t-tests or Wilcoxon signed-rank tests were conducted with *P<0.05, **P<0.01 and ***P<0.001. (B) Cytokine profile of cHL cell lines L1236 and HDLM2. The expression of TNFα, TGFβ and IL10 was analyzed by ELISA (R&D Systems). L1236 showed higher TNFα expression than HDLM2. Data are indicated as mean with SEM of at least 3 independent experiments.
(PDF)

Figure S3 **Effect of GM-CSF and M-CSF on L1236 proliferation and proliferative activity of MΦ in co-culture with L1236.** (A) Co-cultures of L1236 and MΦ were kept in 50% MΦ-conditioned medium containing GM-CSF or M-CSF. Therefore we analyzed the effect of GM-CSF and M-CSF on L1236 growth. L1236 were treated with 25 ng/ml GM-CSF or M-CSF in the presence of BrdU for 48 h. BrdU was stained by an AlexaFluor 488 secondary antibody and fluorescence intensity (FI) was measured by a fluorescence reader. No significant influence of GM-CSF or M-CSF on L1236 proliferation was observed. Mann-Whitney-U-tests were conducted. Data are indicated as mean with SEM of 3 independent experiments. (B) L1236 cells were co-incubated wit MΦ^GM-CSF^ or MΦ^M-CSF^ for 48 h in a ratio of 1∶6 and immunocytochemical doublestainings with Ki67 and CD68 antibodies was conducted. No difference in Ki67 expression in MΦ^GM-CSF^ and MΦ^M-CSF^ was observed. Wilcoxon signed-rank test was conducted. Data are indicated as mean with SEM of 8 independent experiments.
(PDF)
