# Supplementary figures and images for: Macrophages and Dendritic Cells as Actors in the Immune Reaction of Classical Hodgkin Lymphoma
Source: PLoS One. 2014 Dec 3;9(12):e114345. doi: 10.1371/journal.pone.0114345 (PMC4255018; doi:10.1371/journal.pone.0114345)

**Figure S1: Generation of moDC, pro-inflammatory  $M\Phi^{GM-CSF}$  and anti-inflammatory  $M\Phi^{M-CSF}$**

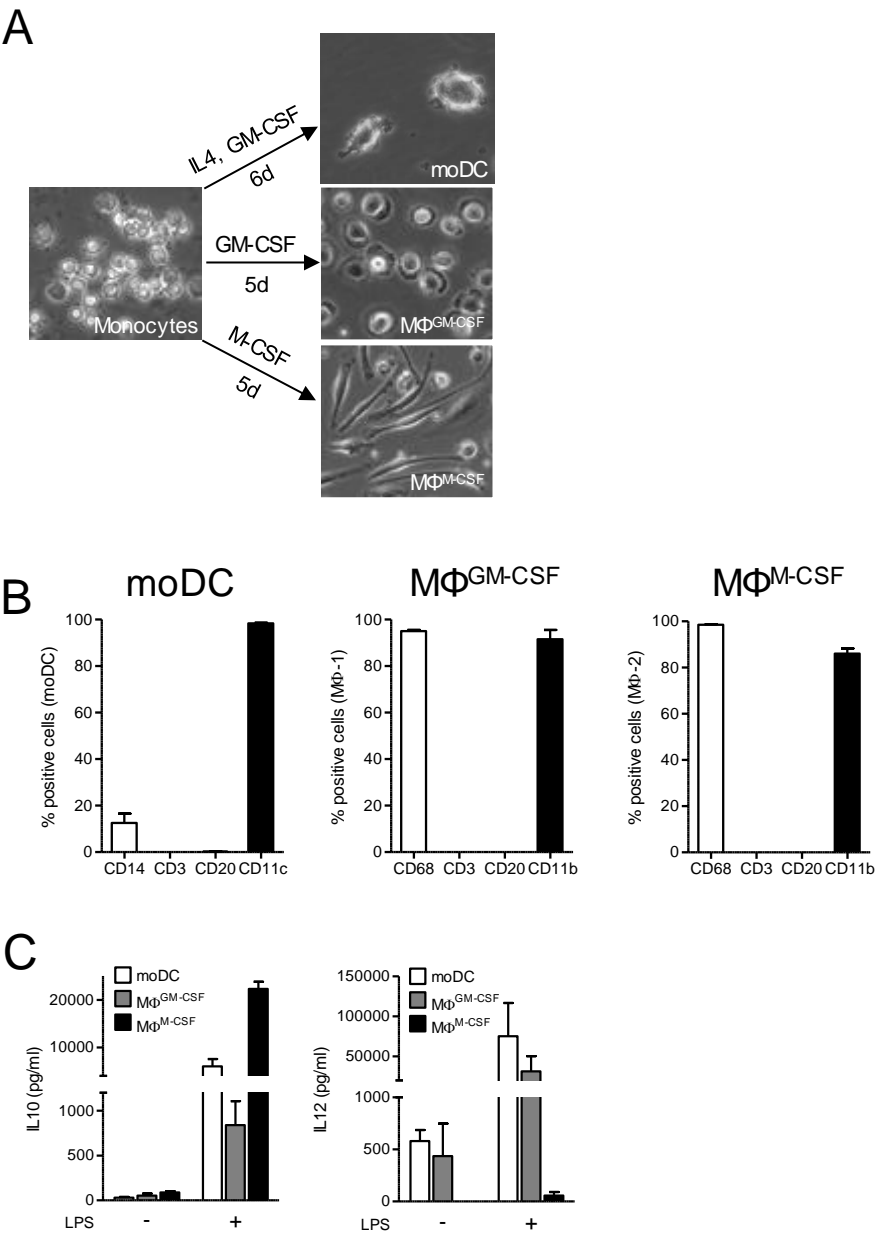

Supplement: Figure S1 — Generation of moDC, pro-inflammatory MΦGM-CSF and anti-inflammatory MΦM-CSF. (A) Human peripheral blood monocytes were differentiated into moDC, MΦGM-CSF or MΦM-CSF in the presence of IL4 + GM-CSF (6 days), GM-CSF (5 days) or M-CSF (5 days), respectively. Depicted are monocytes one day after isolation (left), moDC with branching projections, MΦGM-CSF with a fried-egg morphology and MΦM-CSF with a spindle-shaped morphology. All images were acquired with an Olympus IX81 (100× magnification, 10x/0.30 Ph1 objective) using cellSens dimension software. (B) Purity analysis of moDC, MΦGM-CSF and MΦM-CSF. moDC purity was shown by FACS analysis of CD11c, CD14, CD3 and CD20 (BD Pharmingen) expression, being 98%, 12%, 0% and <1%, respectively. MΦ purity was proven by FACS analysis of CD68, CD11b, CD3 and CD20 (BD Pharmingen). MΦ-1 were positive in 95%, 92%, <1% and <1%, MΦ-2 in 98%, 86%, <1% and <1%, respectively. Data are indicated as mean with SEM of 4 independent experiments. (C) Analysis of cytokine secretion. For the determination of IL10 and IL12 secretion DuoSet ELISAs (R&D Systems) were applied according to the manufacturer's instruction. For generation of supernatants (SN), cells were seeded at a concentration of 1×10^6^/ml+/−100 ng/ml LPS (Sigma-Aldrich) and incubated for 24 h. Absorbance was measured at 450 nm and 540 nm as reference using Synergy 2 (BioTek, Bad Friedrichshall, Germany). Data are shown as mean with SEM of 3–4 independent experiments each. (PDF) [file pone.0114345.s001.pdf]
