# Supplementary material for: Macrophages and Dendritic Cells as Actors in the Immune Reaction of Classical Hodgkin Lymphoma
Source: PLoS One. 2014 Dec 3;9(12):e114345. doi: 10.1371/journal.pone.0114345 (PMC4255018; doi:10.1371/journal.pone.0114345)

Figure S2: Characterization of the maturation and activation profile of moDC and cytokine profile of cHL cell lines L1236 and HDLM2

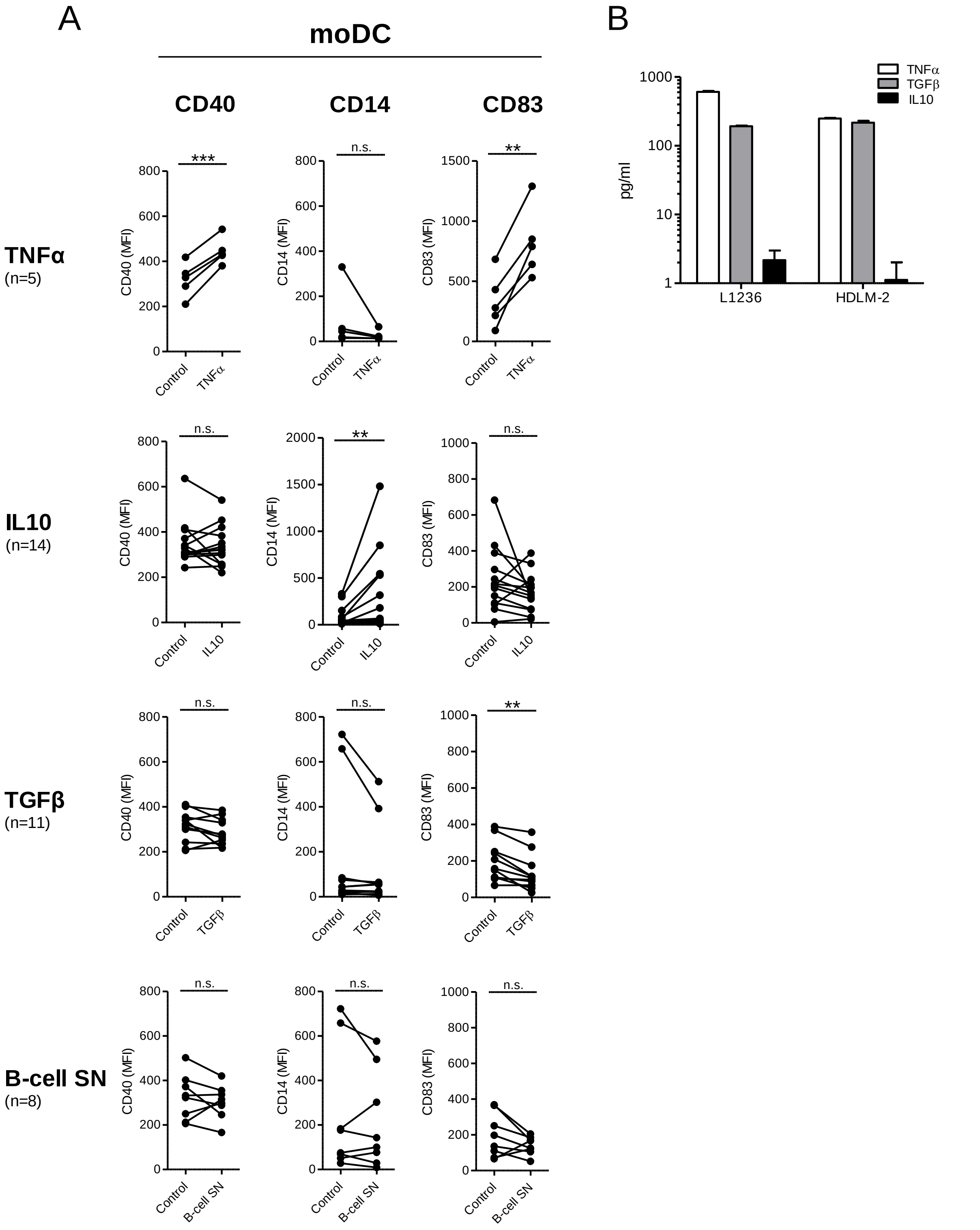

Supplement: Figure S2 — Characterization of the maturation and activation profile of moDC and cytokine profile of cHL cell lines L1236 and HDLM2. (A) moDC were treated for 24 h with 10 ng/ml TNFα, IL10 or TGFβ (each 10 ng/ml) or with 50% B-cell supernatants. B-cells were isolated from human blood PBMC by MACS separation (positive selection) using CD19 MicroBeads, FcR Blocking reagent and MS columns for an OctoMACS separator (MACS Miltenyi Biotec, Bergisch Gladbach, Germany). Supernatants were generated at a concentration of 1×10^6^ cells/ml medium after 48 h incubation. The expression of the DC activation marker CD40, the monocyte marker CD14 and the DC maturation marker CD83 was determined by flow cytometry. As anticipated, TNFα induced the activation marker CD40 and the maturation marker CD83, IL10 counter-acted moDC development as shown by the up-regulation of the monocyte marker CD14, and TGFβ blocked moDC maturation by down-regulation of CD83. SN generated from B-cells from PBMC showed no effect on moDC when compared to medium controls Mean fluorescence intensity (MFI) is indicated on the bar charts. Isotype controls are depicted as dotted lines. Paired student's t-tests or Wilcoxon signed-rank tests were conducted with *P<0.05, **P<0.01 and ***P<0.001. (B) Cytokine profile of cHL cell lines L1236 and HDLM2. The expression of TNFα, TGFβ and IL10 was analyzed by ELISA (R&D Systems). L1236 showed higher TNFα expression than HDLM2. Data are indicated as mean with SEM of at least 3 independent experiments. (PDF) [file pone.0114345.s002.pdf]
