# Supplementary material for: Macrophages and Dendritic Cells as Actors in the Immune Reaction of Classical Hodgkin Lymphoma
Source: PLoS One. 2014 Dec 3;9(12):e114345. doi: 10.1371/journal.pone.0114345 (PMC4255018; doi:10.1371/journal.pone.0114345)

Figure S3: Effect of GM-CSF and M-CSF on L1236 proliferation and proliferative activity of MΦ in co-culture with L1236.

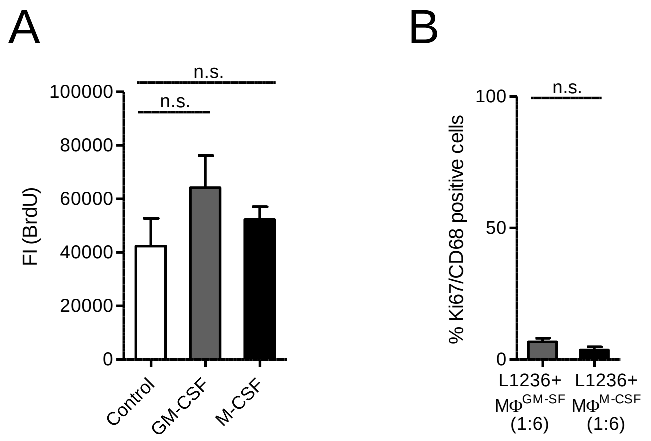

Supplement: Figure S3 — Effect of GM-CSF and M-CSF on L1236 proliferation and proliferative activity of MΦ in co-culture with L1236. (A) Co-cultures of L1236 and MΦ were kept in 50% MΦ-conditioned medium containing GM-CSF or M-CSF. Therefore we analyzed the effect of GM-CSF and M-CSF on L1236 growth. L1236 were treated with 25 ng/ml GM-CSF or M-CSF in the presence of BrdU for 48 h. BrdU was stained by an AlexaFluor 488 secondary antibody and fluorescence intensity (FI) was measured by a fluorescence reader. No significant influence of GM-CSF or M-CSF on L1236 proliferation was observed. Mann-Whitney-U-tests were conducted. Data are indicated as mean with SEM of 3 independent experiments. (B) L1236 cells were co-incubated wit MΦGM-CSF or MΦM-CSF for 48 h in a ratio of 1∶6 and immunocytochemical doublestainings with Ki67 and CD68 antibodies was conducted. No difference in Ki67 expression in MΦGM-CSF and MΦM-CSF was observed. Wilcoxon signed-rank test was conducted. Data are indicated as mean with SEM of 8 independent experiments. (PDF) [file pone.0114345.s003.pdf]
